# Effect of chocolate on older patients with cancer in palliative care: a randomised controlled study

**DOI:** 10.1186/s12904-021-00893-1

**Published:** 2022-01-04

**Authors:** Josiane C. Vettori, Luanda G. da-Silva, Karina Pfrimer, Alceu A. Jordão, Paulo Louzada-Junior, Júlio C. Moriguti, Eduardo Ferriolli, Nereida K. C. Lima

**Affiliations:** Internal Medicine Department, Ribeirão Preto Medical School, University of São Paulo, Avenida Bandeirantes, 3900, Ribeirão Preto, SP 14049-900 Brazil

**Keywords:** Aged, Cancer, Palliative care, Nutritional status, Chocolate

## Abstract

**Background:**

Older advanced stage cancer patients, with changes in nutritional status, represent an important demand for palliative care. The aim was to determine the effects of 4 weeks of chocolate consumption on the nutritional status of older cancer patients in palliative care.

**Methods:**

Older cancer patients in palliative care with ambulatory (*n* = 46) monitoring were randomized to control (CG, *n* = 15), intervention with 55% cocoa chocolate (IG1, *n* = 16) and intervention with white chocolate (IG2, n = 15) groups and evaluated before and after 4 weeks for nutritional status (primary outcome), evaluated by the Mini Nutritional Assessment tool (MNA). Food consumption, anthropometry, body composition, laboratory parameters and quality of life (QL) with the European Organization for the Research and Treatment of Cancer instrument were also evaluated.

**Results:**

IG1 progressed with increased screening (estimated difference [95% CI]: − 1.3 [− 2.2;-0.4], *p* < 0.01), and nutritional (estimated difference [95% CI]: − 1.3 [− 2.5;-0.1], *p* = 0.04) scores on the MNA, with no change in anthropometry and body composition. Regarding antioxidant capacity, reduced glutathione levels increased (estimated difference [95% CI]: − 0.8 [− 1.6;-0.02], p = 0.04) and malondealdehyde levels decreased in IG2 (estimated difference [95% CI]:+ 4.9 [+ 0.7;+ 9.1], *p* = 0.02). Regarding QL, functionality improved in IG1, with higher score in the functional domain (estimated difference [95% CI]:-7.0 [− 13.3;-0.7], *p* = 0.03).

**Conclusions:**

The consumption of chocolate with a greater cocoa content may contribute to the improvement of the nutritional status and functionality among older cancer patients in palliative care. The consumption of white chocolate was associated with improved oxidative stress.

**Trial registration:**

A randomized clinical trial (ClinicalTrials.gov NCT04367493).

## Introduction

Data indicate that, by 2060, about 16 million people per year will die of malignant neoplasias, representing a 109% increase compared to 2016 [1].

This will involve an increase in the number of patients, specially older adults, and their relatives who will need paliiatice care for an appropriate management of the physical, psychosocial and spiritual effects of cancer in order to reduce the suffering and to improve the quality of life (QL) [2].

On this scenario, there is growing concern about the impact of nutrition on cancer patients receiving palliative care. Nutrition should preserve the nutritional status, prevent malnutrition and provide physical, emotional and psychological comfort by rescuing pleasure and convivial memories [3]. Nutritional assistance during palliative care focuses on the most comfortable manner of doing this, respecting food preferences, beliefs and memories [4].

Some foods have been associated with benefits for general well-being, pleasure and emotional comfort [5]. The characteristic flavor, carbohydrate and fat content and highly palatable orosensory qualities of chocolate contribute to its definition as comfort food. Chocolate with a greater cocoa content has beneficial effects, acting against oxidative stress and systemic inflammation, which are risk factors for the proression of cancer [6]. In addition, chocolate can be considered an oral supplementation by being a source of energy and nutrientes, contributing to nutritional requirements [7].

Few studies are available about the impact of nutritional intervention on the QL of patients in palliative care, especially regarding supplements enriched with specific nutrients [8–10], with no studies on accessible consumed foods such as chocolate.

In view of this scenario, the main objective of the present study was to assess the effects of chocolate consumption on the nutritional status of older cancer patients in palliative care. Food consumption, anthropometry, body composition, oxidative stress, inflammatory activity, and QL were also evaluated.

## Methodology

This was a randomised, non-blind clinical trial conducted at the Services of Oncology and Palliative Care of the University Hospital of Ribeirão Preto, University of São Paulo. The study was approved by the Research Ethics Committee of HC-FMRP-USP (Protocol No. 9614/2015) and all subjects gave written informed consent to participate. All methods were performed in accordance with the Declaration of Helsinki and the study was registered on www.clinicaltrials.gov (NCT04367493).

Inclusion criteria: 60 years or older with cancer receiving ambulatory palliative care, with performance status (KPS) ≥60%, > 70% prognosis of 30-day survival according to the Pap Score [11], with no chemotherapy and/or radiotherapy during the last 15 days, normal thyroid function, able to eat orally, and no diagnosis of dementia.

Exclusion criteria: tobacco and/or alcoholic drink use (>3 weekly doses) during the last 3 months, cancer of gastrointestinal location involving the risk of obstructive factors affecting nutrition, and refusal to consume chocolate.

As this study has an unprecedented character, in the sense that it was carried out with older patients under palliative care in the process of disease evolution, which makes it difficult to estimate possible gains in nutritional status, the sample size was defined based on previous randomized studies that performed nutritional intervention with chocolate consumption. Thus, it was observed that the studies already carried out with the consumption of chocolate with nutritional benefits had a total sample number ranging from 11 to 16 patients by the intervention group, whose consumption was carried out for a period of 1 to 30 days [12–15].

**Fig. 1:**
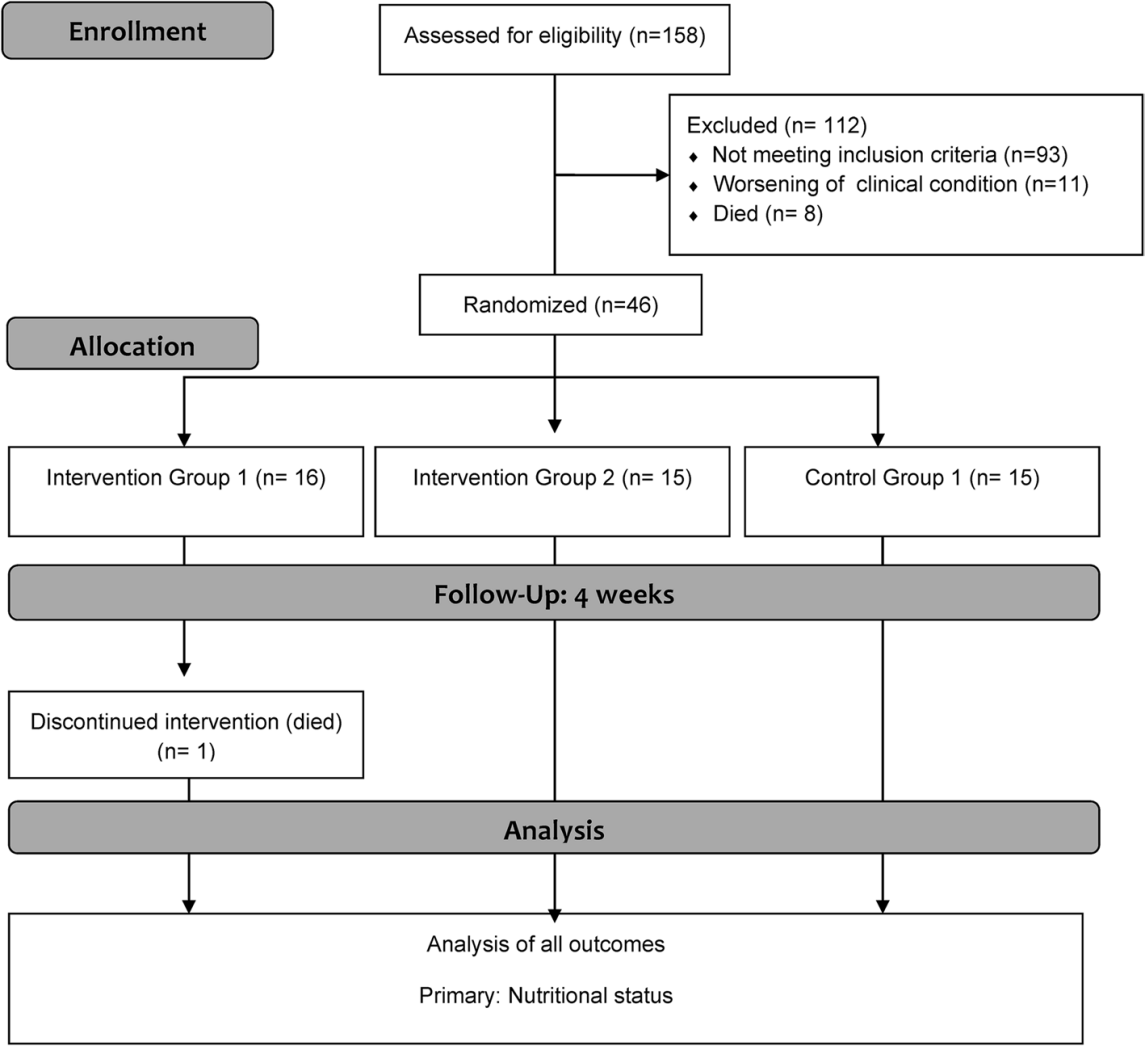
Study flowchart

Subjects were pre-selected (*n* = 156) and 65 were invited to participate. However, 19 were unable to start the protocol. Randomisation was performed using the “Research Randomizer” version 4.0 and 15 participants were included in the control group (CG), 16 in the intervention group receiving 55% cocoa chocolate (IG1), and 15 in the group receiving white chocolate (IG2). One individual of IG1 died due to worsening cancer during the study (Fig. 1). The recruitment and follow-up of participants took place between February 2016 and August 2018.

IG1 patients were instructed to consume 25 g of chocolate containing 55% cocoa daily for 4 weeks, while IG2 consumed 25 g of white chocolate. CG was instructed not to consume extra chocolate, but they could eat other snacks or sweets that they wished. During the study the investigators did not interfere with the habitual food consumption of them, and supplements were maintained. No volunteers had habitual chocolate consumption before the study. Chocolates were supplied in 5 portions of 5 g per day, for a total of 140 tablets.

The chocolate containing 55% cocoa provided a daily amount of 1337 mg polyphenols/ml GAE/patient [16]. IG1 received by day: 126Kcal; 12 g carbohydrates, 1.5 g proteins and 8.8 g total fats, while IG2: 136Kcal; 14 g carbohydrates, 1.4 g proteins and 8.3 g total fats. They recorded daily on a card the amount of consumed chocolate.

All patients were analyzed initially and after 4 weeks with respect to:

- General and health characteristics: sociodemographic and health status data.
- Primary outcome: Nutritional status

The nutritional status was evaluated by the Mini Nutritional Assessment (MNA): a method used for the geriatric population [17] and validated for the Brazilian population [18]. MNA is a screening and diagnostic tool that was built to assess malnutrition, but it also assesses other domains such as mobility, number of medications and cognitive function. This multimodal approach may explain the adequacy of using the MNA to assess the nutritional status of elderly cancer patients in palliative care [19]. In MNA screening, with a maximum of 12 points, the difference of 0.9 point was considered clinically significant [20]. In MNA total assessment, with a maximum of 30 points, the difference of 1.8 points was considered as clinically significant [20].

### Secondary outcomes

1. The 24-h Diet Recall (24HR) and Food Frequency Questionnaire (FFQ): The FFQ was elaborated based on a food list, calibrated [20] and validated for older adults [21], applied only at the end and used to assess the habitual diet consumed during the last 6 months. Nutrient consumption was estimated as: frequency of consumption x portion size x nutritional composition [22]. Food consumed was converted to grams and calculated with the Virtual Nutri Plus software updated with the data of the Brazilian Table of Food Composition [23]. The results obtained were compared to recommended intake of macro- amd micronutrients for the age range [24]. The intake of total polyphenols was quantitated using the Phenol-Explorer databank, version 3.0 [25].
2. Anthropometric evaluation: weight, height, body mass index (BMI) according to the cut-off points for older adults [26], arm circumference (AC), and calf circumference (CC).
3. Body composition: determined by the deuterium oxide method after an 8-h overnight fast. In the morning, each volunteer received 1 ml/kg deuterium oxide (99.9% deuterium oxide, Cambridge Isotope, USA) diluted to 7%, followed by 50 ml natural water for full ingestion of deuterium and mouth washing. Saliva samples were collected before and three hours after intake of the dose. The deuterium enrichment of the samples was determined by isotope ratio mass spectrometry (IRMS, Europa Scientific Hydra System, Cheshhire, UK) after equilbration with 100% hydrogen by the platinum-alumina catalyzer.
4. Routine clinical laboratory tests: blood count, albumin, total proteins, sodium, potassium, and calcium ion.
5. Inflammatory activity: serum levels of interleukin 6 (IL-6) were determined by ELISA with high sensitivity R&D Systems kits (Minneapolis, MN, USA). C-reactive protein was determined by the latex immunoturbidimetric assay.
6. Antioxidant capacity: determination of reduced glutathione (GSH) [27] and ascorbic acid [28] levels.
7. Determination of lipid peroxidation: determination of malondialdehyde (MDA) levels [29].
8. Presence of DNA damage: immunoassay with the DNA/RNA Oxidative Damage EIA Kit (Cayman Chemical) for the detection of all three oxidized quanine species based on 8-hydroxy-2′-deoxyguanosine (8-OHdG) levels.
9. Quality of life: application of the instrument of the European Organization for the Research and Treatment of Cancer (EORTC) - QLQ-C30 Questionnaire [30], with 30 questions including scales of overall health status, symptoms and function, with scores of 0 to 100. The higher these scores, the better the QL. High scores on the symptoms scale indicate a poorer QL (Authorization of the EORTC Quality of Life Group).

Data were analyzed statistically using the SAS Statistical Software, version 9.3 (SAS Institute, Inc. Cary, NC, USA) and the R Core Team (2016).

Data were submitted to descriptive analysis and categorical variables were analyzed by the chi-square test, with the level of significance set at < 0.05. Comparisons of the Mini Nutritional Assessment tool (MNA), food consumption, anthropometry, body composition and other secondary outcomes data were performed using linear mixed model, including random effects that accounted for multiple observations from the same participant (study baseline and end of study) and fixed effect for independence between participants. These models allow for a comparison between the least squared means of the groups at each time point (CG vs. IG1, CG vs. IG2 and IG1 vs. IG2) and comparisons between time points in each group (baseline vs. end), adjusted for age and sex, with corresponding 95% confidence intervals (95%CI). For each model, the assumption of linearity between the relationships was verified graphically, and the residual normality was determined using normal probability plots. The estimated difference (delta) was obtained by the variable of the first group mentioned minus the variable of the second group when different groups were compared, and the baseline minus the end when variables of the same group were compared.

## Results

### Sociodemographic and clinical characterization of the sample

Mean patient age was 67.6 ± 5.7 years (range: 60–83 years) and mean KPS was 88.0 ± 10.9%. Median time of cancer diagnosis was 43.5 months, while median time of diagnosis of locally advanced or metastatic cancer was 11 months.

Mean chocolate consumption was 136 ± 8.3 tablets of 5 g each for IG1 and of 135.8 ± 8.8 tablets for IG2. Groups reported similar and good appreciation of the taste of chocolates. The sociodemographic and clinical characteristics of the patients are listed in Table 1, with no significant difference (*p* > 0.05) between groups.

**Table 1:** Sociodemographic and baseline clinical characteristics of older patients with cancer in palliative care

Variável	CG		IG1		IG2		Full sample	
	n	%	n	%	n	%	n	%
Gender								
Male	6	40	11	69	10	67	27	59
Female	9	60	5	31	5	33	19	41
Ethnicity								
Caucasian	13	87	15	94	11	73	39	85
Mulatto	2	13	1	6	1	7	4	9
Black	0	0	0	0	3	20	3	6
Education								
Illiterate	3	20	0	0	3	20	6	13
Up to 8 years	6	40	13	81	9	60	28	61
9 to 11 years	1	7	2	12	0	0	3	6
More than 11 years	5	33	1	6	3	20	9	20
Marital status								
Single	4	27	0	0	1	7	5	11
Married	6	40	11	69	8	53	25	54
Divorced	2	13	2	12	2	13	6	13
Widower	3	20	3	19	4	27	10	22
Religion								
Catholic	11	73	12	75	7	47	30	65
Evangelical	2	13	3	19	7	47	12	26
Spiritist	1	7	1	6	1	7	3	6
Other	1	7	0	0	0	0	1	2
Occupation								
Retired	11	73	8	50	13	87	32	70
Employee	4	27	8	50	2	13	14	30
Smoking habit								
Former smoker	9	60	9	56	11	73	29	63
Never smoker	6	40	7	43	4	27	17	37
Alcohol abuse								
Drank in the past	2	13	3	18	3	73	8	17
Never drank	13	87	13	81	12	27	38	83
Comorbidities								
Arterial hypertension	5	11	7	15	8	17	20	43
Dyslipidemia	2	4	2	4	2	4	6	13
Depressive disorder	1	2	1	2	3	6	5	11
COPD	0	0	2	4	3	6	5	11
Hypothyroidism	3	6	1	2	0	0	4	9
Renal insufficiency	1	2	2	4	0	0	3	6
Heart disease	1	2	1	2	0	0	2	4
Dementia	0	0	0	0	1	2	1	2
Stroke sequel	0	0	0	0	1	2	1	2
Liver disease	0	0	1	2	0	0	1	2
Other	5	11	3	6	2	4	10	22
Primary tumor site								
Prostate	5	33	6	37	8	53	19	41
Breast	8	53	2	12	3	20	13	28
Lung	2	13	5	31	3	20	10	22
Kidney	0	0	3	19	1	7	4	9
Metastases								
Yes	12	80	16	100	11	73	39	85
Previous oncologic treatment								
Chemotherapy	11	73	13	81	8	53	32	70
Radiotherapy	11	73	4	25	9	60	24	52
Surgery	10	67	10	62	8	53	28	61
Hormone therapy	10	67	6	37	11	73	27	59

*CG* control group, *IG1* intervention group 1 (chocolate with 55% cocoa), *IG2* intervention group 2 (white chocolate), *n* number, % percentage, *COPD* chronic obstructive pulmonary disease

Almost all subjects (93.5%) were taking some type of medications, the more prevalent being antihypertensives (41.3%), nutritional supplements (41.3%), biphosphonates (39.1%), analgesics (37%), antidepressants (23.9%), laxatives (15.2%), and opioids (13%). In CG, 4 patients were using some nutritional supplement: 11% multivitamins, 44% calcium carbonate, 33% vitamin D, 11% vitamin B complex. In GI1, 8 patients: 44% calcium carbonate, 22% vitamin D, 11% ferrous sulfate and 33% powdered nutritional supplement. In GI2, 7 patients were using some nutritional supplement, as follows: 7% multivitamins, 43% calcium carbonate, 43% vitamin D and 7% powdered nutritional supplement.

### Nutritional status

Initially 43.5% of the patients were at risk of malnourished (*n* = 15; 32.6%) or were malnourished (*n* = 5; 10.9%) according to the MAN tool. At the beginning of the study, IG1 patients had a lower score at screening (estimated difference [95% CI]:+ 1.7 [+ 0.5;+ 2.8], *p* < 0.01) and during nutritional assessment (estimated difference [95% CI]:+ 2.1 [+ 0.1;+ 4.1], *p* = 0.04) with the MAN tool compared to CG. IG1 patients showed an increase in the screening (estimated difference [95% CI]:-1.3 [− 2.2;-0.4], p < 0.01) and nutritional assessment (estimated difference [95% CI]:-1.3 [− 2.5;-0.1], p = 0.04) scores at the end of the study (Table 2).

**Table 2:** Anthropometric evaluation, body composition and nutritional status of older patients with cancer in palliative care

VARIABLES	STUDY BASELINE			END OF STUDY		
	Mean ± standard deviation			Mean ± standard deviation		
	CG	IG1	IG2	CG	IG1	IG2
	n = 15	n = 16	n = 15	n = 15	n = 15	n = 15
MNA Screening						
Score (mean ± standard deviation)	11.2 ± 1.4^δ^	9.5 ± 2.4^β^	10.5 ± 1.8	11.6 ± 0.6	10.9 ± 1.2	11 ± 1.5
Nutritional status	n (%)	n (%)	n (%)	n (%)	n (%)	n (%)
Normal	10 (66.7)	9 (56.2)	7 (46.7)	10 (66.7)	8 (53.3)	9 (60.0)
At risk of malnutrition	5 (33.3)	3 (18.7)	7 (46.7)	5 (33.3)	7 (46.7)	6 (40.0)
Malnourished	0 (0)	4 (25.0)	1 (6.7)	0 (0)	0 (0)	0 (0)
MNA Total Assessment						
Score (mean ± standard deviation)	24.7 ± 1.8^α^	22.6 ± 3.9^∞^	23.9 ± 3.1	25.4 ± 1.5	24.7 ± 2.3	25.1 ± 2.5
Nutritional status	n (%)	n (%)	n (%)	n (%)	n (%)	n (%)
Normal	11 (73.3)	9 (60.0%)	9 (60.0%)	12 (80.0)	10 (66.7)	13 (86.7)
At risk of malnutrition	4 (26.7)	5 (33.3)	5 (33.3)	3 (20.0)	5 (33.3)	2 (13.3)
Malnourished	0 (0)	2 (13.3)	1 (6.7)	0 (0)	0 (0)	0 (0)
Anthropometric evaluation						
Body mass index (Kg/m^2^)	29.3 ± 4.5*	26.2 ± 4.0	26.1 ± 3.7	29.3 ± 4.4**	26.3 ± 3.5	26.3 ± 3.8
Arm circumference (cm)	32.4 ± 3.2	30.6 ± 3.5	29.8 ± 3.5	32.2 ± 3.4	30.7 ± 2.8	30.1 ± 3.2
Calf circumference (cm)	37.5 ± 3.0	36.9 ± 3.9	37.5 ± 2.8	37.5 ± 3.1	37.2 ± 3.4	37.3 ± 3.2
Body composition by deuterium						
Total body water (%)	49.3 ± 7.0	48.9 ± 8.1	47.8 ± 7.0	51.5 ± 9.4	48.5 ± 7.4	48.9 ± 5.4
Fat mass (%)	32.7 ± 9.6	33.2 ± 11.1	34.7 ± 9.6	29.6 ± 12.8	33.7 ± 10.1	33.2 ± 7.3
Lean mass (%)	67.3 ± 9.4	66.8 ± 11.1	65.3 ± 9.6	70.4 ± 12.8	66.3 ± 10.1	66.8 ± 7.3

*CG* control group, *IG1* intervention group 1 (chocolate with 55% cocoa), *IG2* intervention group 2 (white chocolate), *n* number, % percentage, *Kg* Kilogram, *MNA* Mini Nutritional Assessment

^δ^ *p* < 0.01 vs. IG1; ^α^ *p* = 0.04 vs. IG1; ^β^ *p* < 0.01 baseline vs. end; ∞ *p* = 0.04 baseline vs. end; * *p* = 0.04 vs. IG1 and IG2; ** *p* = 0.03 vs. IG1

### BMI and body composition

At the beginning, CG had a higher BMI than IG1 (estimated difference [95% CI]:+ 3.0 [+ 0.1;+ 6.0], p = 0.04) and IG2 (estimated difference [95% CI]:+ 3.1 [+ 0.2;+ 6.1], p = 0.04), continuing to be higher than IG1 (estimated difference [95% CI]:+ 3.3 [+ 0.4;+ 6.3], *p* = 0.03) at the end of the study (Table 2). There was no change in body composition in all groups over four weeks time (Table 2).

### Nutritional information of current and habitual food consumption

Estimated data of current and habitual macro- and micronutrient intake are presented in Table 3. At the beginning of the study, the energy and protein intake of more than half the volunteers (*n* = 25; 57.4% and n = 25; 57.4%, respectively) was below the daily recommendations. Mean daily calorie intake was 19.48 ± 4.20 kcal/kg current weight/day and mean protein intake was 0.66 ± 0.22 g /kg current weight /day.

**Table 3:** Description of the nutritional information of current and habitual food consumption of older patients with cancer in palliative care

		24HR STUDY BASELINE Mean ± standard deviation			24HR END OF STUDY Mean ± standard deviation			FFQ Mean ± standard deviation		
	Variables	CG	IG1	IG2	CG	IG1	IG2	CG	IG1	IG2
		n = 15	n = 16	n = 15	n = 15	***n*** = 15	n = 15	n = 15	n = 15	n = 15
	N° of meals**/**day	4.2 ± 0.7	4.4 ± 0.7	4.3	4.0 ± 0.9	4.5 ± 0.7	4.3 ± 0.9	4.3 ± 0.5	4.1 ± 0.4	4.1 ± 0.8
	Kcal/Kg	22.2 ± 6.7	28.4 ± 8.9	25.7 ± 8.9	20.0 ± 6.3	29.6 ± 8.3	25.7 ± 8.3	24.2 ± 6.5	26.3 ± 2.8	25.1 ± 10.1
	Protein/Kg	1.0 ± 0.5	1.2 ± 0.5	1.0 ± 0.5	0.9 ± 0.4	1.3 ± 0.6	1.1 ± 0.5	1.1 ± 0.3	0.9 ± 0.2	1.1 ± 0.5
	Polyphenols (mg)	512.8 ± 163.4	508.7 ± 306.3	513.3 ± 301.2	489.0 ± 225.5	1880.5 ± 414.5	495.6 ± 244.1	577.4 ± 227.1	608.3 ± 168.3	502.6 ± 220.7
	Fiber (g)	16.6 ± 8.8	19.4 ± 9.7	14.2 ± 8.8	16.6 ± 7.8	18.9 ± 9.8	15.1 ± 7.8	26.4 ± 10.4	20.7 ± 12.6	20.6 ± 13.7
% Adequation	Fiber	66.6 ± 32.0	73.5 ± 40.5	50.9 ± 27.5	66.7 ± 27.2	74.3 ± 47.9	55.0 ± 25.0	98.4 ± 32.6	75.8 ± 46.9	71.2 ± 43.0
	Vitamin A	46.6 ± 45.3	73.1 ± 85.3	38.9 ± 52.9	39.2 ± 32.7	61.2 ± 63.1	36.1 ± 37.2	63.9 ± 67.7	28.6 ± 34.1	31.9 ± 40.7
	Vitamin B6	47.0 ± 39.2	82.5 ± 96.0	57.0 ± 41.7	43.2 ± 21.8	70.0 ± 44.5	41.9 ± 33.4	26.6 ± 13.6	35.4 ± 19.3	38.3 ± 23.9
	Vitamin B12	56.9 ± 62.8	43.5 ± 72.7	17.8 ± 24.7	17.2 ± 16.6	121.6 ± 158.5	149.3 ± 312.9	35.1 ± 29.2	51.8 ± 46.5	54.6 ± 53.4
	Vitamin C	202.4 ± 411.9	134.1 ± 153.3	74.5 ± 94.4	99.0 ± 96.0	139.1 ± 137.9	108.9 ± 108.6	170.9 ± 136.5	165.2 ± 141.9	126.8 ± 121.5
	Vitamin E	56.6 ± 41.5	98.3 ± 87.8	47.6 ± 38.5	54.9 ± 41.7	73.8 ± 62.4	73.1 ± 48.8	26.5 ± 31.9	44.9 ± 62.6	29.4 ± 29.3
	Calcium	33.6 ± 28.1	40.7 ± 15.6	46.2 ± 35.6	35.4 ± 21.5	53.9 ± 29.2	36.7 ± 24.4	44.1 ± 11.9	41.6 ± 16.6	35.3 ± 18.7
	Sodium	178.4 ± 69.7	208.0 ± 85.4	188.5 ± 43.3	177.9 ± 55.8	209.2 ± 72.8	194.2 ± 73.5	191.1 ± 49.6	191.2 ± 19.0	193.8 ± 36.0

*CG* control group, *IG1* intervention group 1 (chocolate with 55% cocoa), *IG2* intervention group 2 (white chocolate), *24HR* 24-h Diet Recall, *FFQ* Food Frequency Questionnaire, % percentage, n° number, *Kcal* Kilocalorie, *Kg* Kilogram, *mg* miligram, *g* gram

Calorie consumption (Kcal/kg weight) was lower in CG after the intervention period (estimated difference [95% confidence interval (CI)]: 2.2 [+ 0.5 to + 3.9]; *p* = 0.01). Group comparison also showed that calorie consumption was lower in CG than in IG1 both at the beginning (estimated difference [95% CI]: − 6.1 [− 11.9;-0.4], *p* = 0.04) and at the end (estimated difference [95% CI]: − 9.3 [− 15.1;-3.5], *p* < 0.01) of the study.

At the end of the study, polyphenol consumption increased in IG1 (estimated difference [95% CI]: − 1356.1 [− 1480.2;-1233], *p* < 0.01) and was higher than in CG (estimated difference [95% CI]:-1375.8 [− 1586.2;-1165.4], *p* < 0.001) and IG2 (estimated difference [95% CI]:+ 1369.2 [+ 1158.8;+ 1579.6], p < 0.01).

Except for sodium, the intake of fibers, calcium and of all vitamins analyzed (A, B6, B12, C and E) was below recommended levels.

### Laboratory exams and QL

Table 4 presents the results of the laboratory tests. After the 4 weeks, there was an increase in 8–OHdG in all groups: CG (estimated difference [95% CI]:-1.3 [− 2.2;-0.4], *p* < 0.01), IG1 (estimated difference [95% CI]:-1.1 [− 2.0;-0.3], p < 0.01) and IG2 (estimated difference [95% CI]:-0.9 [− 1.8;-0.02], *p* = 0.04).

**Table 4:** Description of the results of laboratory tests of older patients with cancer in palliative care

VARIABLES	STUDY BASELINE			END OF STUDY		
	Mean ± standard deviation			Mean ± standard deviation		
	CG	IG1	IG2	CG	IG1	IG2
	n = 15	n = 16	n = 15	n = 15	n = 15	n = 15
**Hemoglobin** (U/dL)	13.0 ± 1.3	12.7 ± 1.8	12.5 ± 2.1	13.0 ± 1.4*	12.3 ± 1.8	12.6 ± 1.5
**White blood cells** (×103/μL)	5.9 ± 2.0	7.3 ± 3.4	5.7 ± 1.8	6.0 ± 2.0	6.08 ± 2.0	7.0 ± 3.2
**Lymphocytes** (×103/μL)	1.5 ± 0.4	1.9 ± 0.6	1.8 ± 0.7	1.6 ± 0.5	1.68 ± 0.5	1.7 ± 0.5
**Total proteins** (U/dL)	6.8 ± 0.5	6.7 ± 0.7	6.9 ± 0.6	6.9 ± 0.4	6.69 ± 0.7	7.0 ± 0.7
**Albumin** (U/dL)	4.2 ± 0.3*	4.0 ± 0.4	4.1 ± 0.3	4.2 ± 0.2*	4.0 ± 0.3	4.2 ± 0.3
**Vitamin** (mg/dL)	0.3 ± 0.1**	0.2 ± 0.1	0.2 ± 0.1	0.4 ± 0.1**	0.2 ± 0.1	0.2 ± 0.1
**C-reactive protein** (mU/dL)	0.4 ± 0.5	2.4 ± 4.0	1.8 ± 3.4	0.9 ± 1.2	2.4 ± 3.0	1.4 ± 3.2
8-OHdG (ng/mL)	4.6 ± 2.2 ^δ^	4.9 ± 2.2 ^δ^	4.4 ± 1.6 ^β^	5.9 ± 2.6	6.0 ± 2.0	5.3 ± 1.4
MDA (μM)	10.9 ± 3.3	14.4 ± 10.2	15.6 ± 11.9 ^α^	9.1 ± 2.6	11.3 ± 3.0	10.8 ± 4.8
GSH (μM)	9.8 ± 1.4	9.0 ± 1.8	10.0 ± 11.1 ^β α^	10.3 ± 2.0	8.8 ± 1.5^∞^	10.7 ± 2.1
Interleukin 6 (pg/mL)	51.8 ± 38.6	136.6 ± 182.3	65.21 ± 132.49	87.2 ± 125.6	154.8 ± 208.4	38.7 ± 25.0

*CG* control group, *IG1* intervention group 1 (chocolate with 55% cocoa), *IG2* intervention group 2 (white chocolate), *8-OGdG* 8-hydroxy-2′-deoxyguanosine, *MDA* malondialdehyde, *GSH* reduced glutathione, *μL* microliter, *U* unit, *dL* deciliter, *mU* miliunit, *mg* miligram, *μM* micromol, *pg* picogram, *mL* milliliter

* *p* = 0.03 vs. IG1; ** *p* < 0.01 vs. IG1 e IG2; ^δ^ *p* < 0.01 baseline vs. end; ^α^ *p* = 0.02 baseline vs. end; ^β^ *p* = 0.04 baseline vs. end; ^∞^ *p* = 0.02 vs. CG e < 0.01 vs. IG2

Regarding the antioxidant capacity, GSH levels were lower in IG1 than in CG (estimated difference [95% CI]:1.6 [+ 0.2;+ 2.9], *p* = 0.02) and IG2 (estimated difference [95% CI]:-2.1 [− 3.4;-0.7], p < 0.01) at the end, with an increase in IG2 (estimated difference [95% CI]:-0.8 [− 1.6;-0.02], p = 0.04). In contrast, vitamin C levels were lower in the intervention’s groups than in CG at the beginning (estimated difference [95% CI]:+ 0.1 [+ 0.07;+ 0.2], *p* < 0.01) between IG1 and CG; (estimated difference [95% CI]:+ 0.1 [+ 0.08;+ 0.2], p < 0.01) between IG2 and CG and at the end of the study (estimated difference [95% CI]:+ 0.2 [+ 0.1;+ 0.3], p < 0.01) between IG1 and CG; (estimated difference [95% CI]:0.2 [+ 0.1;+ 0.3], p < 0.01) between IG2 and CG.

Lipid peroxidation, with MDA levels, was reduced in IG2 (estimated difference [95% CI]:+ 4.9 [+ 0.7;+ 9.1], p = 0.02) from the beginning to the end and IL-6 levels were higher in IG1 (estimated difference [95% CI]:+ 116.1 [+ 12.9;+ 219.3], *p* = 0.03) than in IG2 at the end of the study.

The QL of IG1 patients (Table 5) improved in terms of functionality, with a higher score for the functional domain (estimated difference [95% CI]:-7.0 [− 13.3;-0.7], p = 0.03), the role functioning subdomain (estimated difference [95% CI]:-21.4 [− 36.4;-6.3], p < 0.01), and the social subdomain (estimated difference [95% CI]:-16.8 [− 28.8;-4.8], *p* < 0.001).

**Table 5:** Score of quality of life domains of older patients with cancer in palliative care

DOMAINS	STUDY BASELINE			END OF STUDY		
	Mean ± standard deviation			Mean ± standard deviation		
	CG	IG1	IG2	CG	IG1	IG2
	n = 15	n = 16	n = 15	n = 15	n = 15	n = 15
Global health status	83.9 ± 16.2	79.7 ± 12.9	75.6 ± 17.1	79.4 ± 23.1	83.3 ± 11.4	82.2 ± 16.3
Functional	83.6 ± 12.4	75.6 ± 17.3 ^β^	82.1 ± 14.2	82.1 ± 13.5	82.7 ± 8.9	83.1 ± 17.0
Physical functioning	84.0 ± 15.3	72.5 ± 19.2	84.9 ± 16.4	84.9 ± 18.4	80.0 ± 18.0	82.7 ± 22.4
Role functioning	94.4 ± 12.1*	60.4 ± 37.5**^∞^	82.2 ± 24.8	82.2 ± 29.9	83.3 ± 20.9	88.9 ± 24.1
Emotional functioning	77.8 ± 28.1	82.8 ± 25.4	74.4 ± 23.2	73.9 ± 30.5	78.9 ± 16.9	75.0 ± 27.6
Cognitive functioning	78.9 ± 23.1	86.5 ± 17.5	82.2 ± 24.0	87.8 ± 14.7	90.0 ± 12.3	87.8 ± 18.3
Social functioning	87.8 ± 24.8	72.9 ± 28.5 ^δ∞^	90.0 ± 18.7	85.6 ± 27.4	88.9 ± 13.6	90.0 ± 16.4
Symptom	9.6 ± 9.5 ^α^	18.3 ± 11.6	14.0 ± 14.4	12.0 ± 12.0	14.9 ± 10.2	12.0 ± 13.5

*CG* control group, *IG1* intervention group 1 (chocolate with 55% cocoa), *IG2* intervention group 2 (white chocolate)

* *p* < 0.01 vs. IG1; ** *p* = 0.03 vs. IG2; ^δ^ *p* = 0.04 vs. IG2; ^α^ *p* = 0.05 vs. IG1; ^β^ *p* = 0.03 baseline vs. end; ^∞^ *p* < 0.01 baseline vs. end

There was no deleterious effect that could be attributed to the consumption of dark or white chocolate, such as nausea, vomiting, diarrhea, or epigastric pain.

## Discussion

The present study, conducted on older cancer patients in palliative care with preserved functionality, demonstrated benefits in terms of improved nutritional status and QL in the group ingesting chocolate with a higher percentage of cocoa. IG1 showed an increased estimated polyphenol intake at the end of the intervention compared to CG and IG2. Several studies that used the values of the Phenol-Explorer databank or values measured by HPLC have reported a daily polyphenol intake ranging from 377 ± 15 to [31] 1756.5 ± 695.8 [32] mg/day in many countries [31–33]. However, all studies were conducted on healthy subjects, with no study on palliative care cancer patients. Considering that the mean worldwide intake of polyphenols is approximately 1 g/day, the present study detected a habitual daily intake of two to three times less, in agreement with the result reported in a Brazilian population study [31].

According to the MAN nutritional screening, most participants had an adequate nutritional status both at the beginning and at the end of the study. Previous studies have reported higher proportions of malnutrition among cancer patients in palliative care. However, those studies were more heterogeneous regarding the primary location of the tumor, nutritional assessment methods, and funcionality [34–36]. This divergence may be attributed to the inclusion criteria of the present study.

At the beginning of the study, IG1 subjects had lower screening and nutritional assessment scores determined by the MAN tool and lower BMI and albumin values compared to the other groups. However, at the end of the intervention period, their screening score and MAN results were increased. The elevation of the scores had clinical significance, with no individual in the classification of malnourished after 4 weeks in IG1. Nutritional intervention can reduce the weight loss of patients in an advanced stage of cancer and improve their nutritional status [37].

No differences in body composition were observed between groups, possibly owing to the short period of intervention. Nevertheless, it should be pointed out that changes in body composition in response to changes in the metabolic demand, physiological changes, aging and alterations due to cancer treatment are frequent among older adults receving palliative care and should be monitored [38].

Except for sodium, the intake of fibers, calcium and of all vitamins analyzed (A, B6, B12, C and E) was below recommended levels. With aging and progression of oncologic disease, modifications may occur in food consumption due to factors such as loss of appetite, sensory changes in gustatory and olfactory capacity, and social, emotional and economic aspects such as social isolation and depression, with a consequent reduction of the intake and absorption of micronutrients essential for health [39, 40].

However, the opposite was observed regarding sodium intake, which was excessive in all groups. This result has been associated with the increased consumption of processed and ultraprocessed foods by the population [36], with 80% of Brazilian older males and 61% of Brazilian older females habitually consuming higher than recommended sodium amounts [41].

Regarding energy and protein consumption, at the beginning and at the end of the study, more than half the patients had lower than recommended.

At the beginning and at the end of the study, CG showed lower calorie consumption per Kg than IG1 even with a higher BMI, a higher MAN score and albumin value, and better functionality. Despite the difficulty in interpreting this finding, we believe that IG1 had a greater consumption per Kg as a form of compensation for its worse basal nutritional status. On the other hand, it has been demonstrated that reduced food intake or low energy intake is independently associated with weight loss in oncologic patients during progression of the disease [42, 43].

Laboratory work-up demonstrated progression of oncologic disease. 8-OHdG levels were significantly increased in all groups, being possibly associated with the evolution of cancer patients [44].

After the period of intervention, IG1 showed an increase in the levels of the proinflammatory cytokine IL-6 with a concomitant reduction of the antioxidant defense compared to the other groups. These results suggest a worse clinical situation of these patients who already showed greater nutritional impairment at the beginning of the study. Systemic inflammation is associated with worse clinical outcomes, including reduced survival, of cancer patients [45]. GSH and vitamin C play a prominent role in cell protection against cytotoxic and carcinogenic substances [46].

Oxidative stress activates the inflammatory pathways that lead to the transformation of a normal cell into a neoplastic one, also affecting survival, proliferation, invasion, angiogenesis, and resistance to oncologic treatment [47]. Conversely, there is evidence that circulating IL-6 levels may also affect the antioxidant defense system [48]. During the final phase of the study, IL-6 levels were found to be significantly lower in IG2 compared to IG1. In agreement, the levels of MDA, a product of lipid peroxidation, were significantly reduced and GSH was increased in the white chocolate group.

We believe that the beneficial action of white chocolate consumption on systemic inflammation and the defense against oxidative stress may be the effect of some not yet studied component. The benefits of white chocolate intake were also observed in a study by OSTERTAG et al. (2013) [49] conducted on healthy subjects, showing that the consumption of 60 g of white chocolate in a single intake contributed favorably to platelet activation and to bleeding time compared to bitter chocolate. Since white chocolate does not contain flavonoids, the authors suggested that other compounds such as milk serum protein may be responsible for antiplatelet effects [49]. Thus, we may consider white chocolate not to have a placebo effect, except for the evaluation of the polyphenol consumption.

Regarding the QL of the patients, IG1 progressed to higher scores in the functionality domain and subdomains, suggesting that the consumption of chocolate with a higher cocoa content was of benefit in terms of QL. We considered that the improvement had clinical significance, as the increase in the score was greater than 10 in a total of 100.

In a previous study, the authors observed low scores on global and functional health scales, with role functioning showing the worst evaluation, as well as high scores on the symptom scale [50]. In the present srudy, volunteers showed a good QL by the global health scale and role functiong score, and the initial symptom score was low.

Few studies have analyzed the effect of dark chocolate consumption on QL, but publications suggest that supplementation with high cocoa chocolate can be of benefit [51, 52].

### Strengths and limitations of the study

This is a randomized, controlled study of nutritional intervention with chocolate. To date, we have not found any other studies that evaluated this intervention in elderly people with cancer in palliative care. The results obtained may be applicable to patients in conditions like those studied. The limitations of the present study were a small number of subjects and a short period of intervention. However, this is an inherent difficulty of clinical studies in palliative care. We suggest that further interventions should explore the relations and the underlying causal mechanisms regarding chocolate consumption and its effects on the health and QL of older patients on palliative care.

## Conclusions

The present results demonstrate that the consumption of chocolate with a higher cocoa content may contribute to improved nutritional status and functionality among older cancer patients in palliative care with > 70% prognosis of 30-day survival. The consumption of white chocolate was associated with an improvement of oxidative stress parameters.

Good adherence to the consumption of both chocolate types was observed during the study, this being a viable and pleasurable food of easy access contributing to the food supply and well-being of the patients.

Considering that food preferences are highly personal, we believe that nutritional support should also be adapted to the necessities, wishes and preferences of everyone in order to be effective and applicable to the reality of each one. In this respect, nutritional assistance can be an opportunity to aid the patients and their families during treatment.

## Data Availability

The datasets used and/or analysed during the current study are available from the corresponding author on reasonable request.
